# Recent developments in palifermin basic, pre-clinical and clinical research

**DOI:** 10.1111/jcmm.12132

**Published:** 2013-09-23

**Authors:** Jeffrey S Rubin

**Affiliations:** Potomac, MD, USA

Keratinocyte growth factor (KGF or FGF7) was originally identified as an epithelial-specific mitogen of mesenchymal origin [1]. Its target cell specificity was attributed to the epithelial distribution of alternatively spliced FGFR2b transcripts, which encode the only receptors that bind KGF with high affinity [2, 3]. Initially, KGF was thought to be a key paracrine effector of mesenchymal–epithelial communication during development. Consistent with this view, the disruption of FGFR2b expression resulted in abnormal organogenesis that featured major defects in branching morphogenesis [4]. However, subsequent work showed that another FGF family member with high affinity for FGFR2b, FGF10, was the critical mediator of FGFR2b signalling during development [5]. Knockout of KGF expression had relatively subtle effects on development that were evident in hair, kidney and bladder (reviewed in [6]). In contrast, KGF was markedly up-regulated following injury [7, 8], and healing was significantly impaired in mice lacking KGF expression [9]. These and other observations led to the conclusion that KGF had an important role in the repair of epithelial tissues.

Palifermin is a recombinant, amino-terminally truncated derivative of KGF with stable properties and high yields when expressed in bacteria. A large number of pre-clinical studies have demonstrated that it has protective effects on many epithelial tissues treated with a variety of noxious agents, particularly when administered prior to the toxic exposure [6]. Its beneficial effects are because of multiple mechanisms, including stimulation of cell proliferation, migration, differentiation, survival, DNA repair and induction of enzymes that inactivate reactive oxygen species. A programme was initiated to investigate its use in limiting damage to the oral mucosa that results from intensive cancer chemo/radiotherapy. Oral mucositis is a painful, debilitating side-effect of many cancer treatment protocols that can lead to medical complications, delays in therapy and increased cost [10]. In 2004, the U.S. Food and Drug Administration approved the use of palifermin to decrease the incidence and duration of severe oral mucositis in patients with haematological malignancies who receive intensive cytotoxic therapy that requires haematopoietic stem-cell support.

Following this regulatory approval, ongoing efforts have explored other potential applications of palifermin. The article by Finch *et al*. summarizes an array of research pertaining to the basic biology of KGF/FGFR2b and pre-clinical models that suggest additional areas for clinical development. Many of these studies have incorporated novel ways to deliver the drug, such as gene transfer strategies or impregnation of palifermin protein in matrices, to increase its efficacy and reduce side-effects. Other positive results have led to phase I and phase II clinical trials to test the safety and efficacy of palifermin in patients with acute lung injury. Recent work also indicates that KGF may have relevance beyond the realm of mesenchymal–epithelial interactions and tissue repair, as it appears to function as a pre-synaptic organizing molecule in the brain. The differential expression of KGF and related FGF family members, as well as dysregulation of FGFR2b, may contribute to a number of neurological disorders.

The article by Vadhan-Raj *et al*. provides an account of the palifermin clinical trials that led to its regulatory approval and a review of many subsequent trials that have further explored its use in patients with cancer. In the landmark phase III trial involving patients with haematological malignancies who received autologous haematopoietic stem-cell transplants, palifermin was given before and after a conditioning regimen consisting of total body irradiation and high-dose chemotherapy [11]. Additional trials have investigated its utility in patients treated with less toxic regimens. Based on the cytoprotective effects of KGF on the thymic epithelium that promoted T-cell maturation in animal studies, clinical trials are being conducted to determine whether palifermin can expedite the reconstitution of the immune system following transplantation. Such an effect would decrease the risk of infection and tumour relapse faced by transplant recipients. Pre-clinical work also suggested that palifermin might limit graft-versus-host disease after allogeneic transplantation, and clinical trials have been designed to test this possibility. At the same time, others have been investigating the safety and efficacy of palifermin in patients with solid tumours. Here, questions arise regarding the possibility that palifermin activity on tumour cells expressing its receptor might undermine cancer therapy [12, 13]. Thus far, there is no evidence that palifermin adversely affects patient response to therapy, but this issue continues to be closely monitored. In principle, palifermin might prolong survival by enabling patients to tolerate more effective treatment regimens that otherwise would be too toxic. With increasing experience in the clinic, it has become apparent that the dosing schedule for palifermin used in the phase III haem transplant trial is not optimal or practical in a number of other treatment protocols. As discussed by Vadhan-Raj and colleagues, a flexible approach to palifermin dosing will be necessary to maximize the likelihood of its successful incorporation into different treatment regimens. Taken together, the two articles in this series present a thorough review of the opportunities, progress and challenges in developing new applications for palifermin.
